# The Effect of Extra Virgin Olive Oil and Soybean on DNA, Cytogenicity and Some Antioxidant Enzymes in Rats

**DOI:** 10.3390/nu6062376

**Published:** 2014-06-23

**Authors:** Thanaa A. El-Kholy, Mohammad Abu Hilal, Hatim Ali Al-Abbadi, Abdulhalim Salim Serafi, Ahmad K. Al-Ghamdi, Hanan M. Sobhy, John R. C. Richardson

**Affiliations:** 1 Department of Clinical Nutrition, Faculty of Applied Medical Sciences, King Abdulaziz University, P.O. Box 80200, Jeddah 21589, Saudi Arabia; 2 School of Medicine, University of Southampton, Southampton SO16 6YD, UK; E-Mail: abuhilal9@gmail.com; 3 Experimental Surgery Unit, KFMRC (King Fahd Medical Research Center), Faculty of Medicine, King Abdulaziz University, Jeddah 21589, Saudi Arabia; E-Mail: hatimalabbadi@yahoo.com; 4 Faculty of Medicine, Umm Al Qura University, Makkah 21955, Saudi Arabia; E-Mail: abdulhalims@hotmail.com; 5 Department of Medical Laboratory Technology, Faculty of Applied Medical Sciences, King Abdulaziz University, P.O. Box 80200, Jeddah 21589, Saudi Arabia; E-Mail: aalghamdi@kau.edu.sa; 6 Pharmacology Unit, Biochemistry, Toxicology and Food deficiency Department, Animal Health Research Institute, Dokki, Gizza, Egypt; E-Mail: dr.hanan_mss@yahoo.com; 7 Surgical Registrar, University Hospital Southampton, Southampton SO16 6YD, UK; E-Mail: johnrcr@hotmail.com

**Keywords:** soybean, olive oil, supplements, rats

## Abstract

We investigated the effect of extra virgin (EV) olive oil and genetically modified (GM) soybean on DNA, cytogenicity and some antioxidant enzymes in rodents. Forty adult male albino rats were used in this study and divided into four groups. The control group of rodents was fed basal ration only. The second group was given basal ration mixed with EV olive oil (30%). The third group was fed basal ration mixed with GM (15%), and the fourth group survived on a combination of EV olive oil, GM and the basal ration for 65 consecutive days. On day 65, blood samples were collected from each rat for antioxidant enzyme analysis. In the group fed on basal ration mixed with GM soyabean (15%), there was a significant increase in serum level of lipid peroxidation, while glutathione transferase decreased significantly. Interestingly, GM soyabean increased not only the percentage of micronucleated polychromatic erythrocytes (MPCE), but also the ratio of polychromatic erythrocytes to normochromatic erythrocytes (PEC/NEC); however, the amount of DNA and NCE were significantly decreased. Importantly, the combination of EV olive oil and GM soyabean significantly altered the tested parameters towards normal levels. This may suggest an important role for EV olive oil on rodents’ organs and warrants further investigation in humans.

## 1. Introduction

Plant food represents a major source of nutrients for humans. As well as performing a protective role against chronic diseases such as coronary heart disease, diabetes mellitus and cancer [1]; plants are a good source of omega-3 fatty acids, they are rich in antioxidant vitamins (such as alphatocopherol, ascorbic acid and beta-carotene), glutathione, proteins and advantageously they can be grown in many environments including arid climates [2].

Extra virgin (EV) olive oil is a vegetable oil obtained from olive trees (oleaeuropaea); a traditional tree crop of the Mediterranean Basin. It has many applications including cooking, cosmetics, pharmaceutical preparations and soaps. Olive oil is considered a healthy product because of its constituents, which include oleic acid, palmitic acid and other fatty acids; in addition to traces of squalence and sterols. There is considerable data demonstrating that the consumption of olive oil is beneficial to cardiovascular health; specifically it has a favorable effect on cholesterol regulation and LDL cholesterol oxidation. It has also been shown to have anti-inflammatory, antithrombotic, antihypertensive and vasodilatory effects in both animals and humans [3].

Soyabean is considered to be one of the most important crops worldwide. It is generally considered to be a protein concentrate as this represents 42% of its make up [4]. Industrially, it is an essential food component for humans and for a wide range of animals [1].

Ram *et al.* [5] found that soyabean contains large amounts of protein and fiber, while Li *et al.* [6] reported hypersensitivity type reactions in weaned pigs and in some cases death when fed soyabean meal only. Importantly, Fatma [7] reported an increased percentage of death and fetal abnormalities associated with soybean consumption in rats. Furthermore, genetically modified (GM) soya bean may also induce damage to DNA in rats, resulting in an acute hepatitis picture that resembles human viral hepatitis [8,9].

The primary aim of this study was to elucidate any potential hepato-protective role that may be performed by natural products such as GM soyabean and whole EV olive oil. To accomplish this we examined their anti-oxidative effects, both individually and in combination, by investigating their effects on damage to DNA in rats. Interestingly, we found a possible protective role when animals are fed with a mixture of EV olive oil and GM soya bean and we believe this outcome represents an important opportunity to examine the potential benefits of these valuable natural products further.

## 2. Material and Methods

### 2.1. Animals

Forty adult white albino rats (Sprague Dawley strain) with an average weight of (150 to 180 g), were used in this study. They were obtained from the laboratory animal house of the ophthalmic research institute, Giza, Egypt. The rats were acclimatised to laboratory condition before the experiment began. They were fed according to the experimental design detailed later in the manuscript and supply was given ad-libitum. All experiments were conducted according to the University’s ethical protocols.

### 2.2. Plant Material

Soyabean: Soyabean grind was purchased from a local market in Cairo, Egypt. It was freshly prepared and mixed with basal ration for rats, at a concentration of 15% [10].

Extra virgin olive oil: was purchased from a local market in Cairo, Egypt. It was freshly prepared and mixed with basal ration for rats, at concentration of 30% [11]. The chemical composition of these is detailed in Table 1.

**Table 1:** Feeding structure.

Group	Ration
Group 1	The negative control group was fed basal ration diet only.
Group 2	Fed basal ration diet mixed with EV olive oil (30%).
Group 3	Fed basal diet mixed with GM soyabean (15%).
Group 4	Fed basal diet mixed with EV olive oil (30%) and GM soyabean (15%).

### 2.3. Experimental Design

Forty adult rats were divided into four groups of ten. The experiment was conducted over 65 consecutive days. The feeding structure for each group is detailed in Table 1.

### 2.4. Sampling

#### 2.4.1. Serum Samples

Blood samples were obtained from each animal group from their orbital plexuses and received into a clean, dry tube. Samples were left to clot at room temperature for 2 h, stored overnight in a refrigerator at 4 °C and centrifuged at 3000 revolutions per minute (rpm) for 15 min. Serum samples were then drawn in clean, dry capped bottles and kept in deep freeze at 4 °C for antioxidant enzyme studies. (The animals were fasted for 12 h prior to the sacrificing).

#### 2.4.2. Antioxidant Enzymes Estimation

Lipid peroxide was obtained using thiobarbitutric acid reaction outlined by Ohkawa *et al.* [12] and purification of Glutathion-transferase was achieved according to Habig *et al.* [13].

#### 2.4.3. Determination of DNA

The isolation of DNA from spleen of rats was performed according to the following protocol: first, the tissue was homogenized in isotonic saline buffered with sodium citrate (pH 7.0); protein was then removed by treatment with a chloroform/amyl alcohol mixture and the DNA precipitated with ethanol according to protocols established by Schwander *et al.* [14]. Estimation of isolated DNA was carried out using diphenylamine reagent and read at 595 nm on spectrophotometer according to the method described by Dische *et al.* [15].

#### 2.4.4. Cytogenicity

The micronucleus test was performed to detect chromosomal damage associated with treatment. Micronuclei were identified as dark blue staining bodies in the cytoplasm of the polychromatic erythrocytes (PCEs) according to the protocol mentioned by Salamon *et al.* [16]. The polychromatic erythrocytes (PCEs, 1000/animal) were screened for micronuclei and the changes in the mitotic activity were assessed on the basis of the ratio of polychromatic to normochromatic erythrocytes (PCE/NCE ratio) [17,18].

### 2.5. Statistical Analysis

Parametric data were interpreted using the analysis of variance (ANOVA) test and comparative of means were performed using the Duncan Multiple Range test [19] using SPSS 14 [20].

## 3. Results

This study was carried out to investigate the effects of EV olive oil and GM soybean on DNA cytogenicity, as well as looking into its antioxidant role. To address these questions male albino rats were fed one concentration of each plant alone, or combined, for 65 days. We examined the antioxidant enzymes analyses and investigated in order to determine the DNA damage in the spleens and its cytogenicity, in rats.

Diet effects on antioxidant enzymes: Feeding on ration mixed with GM soyabean at concentration 15% caused significant increase in serum level of lipid peroxidation, while the level of glutathione dehydrogenase was significantly reduced (Table 2).

Ration mixed with EV olive oil and GM soyabean significantly altered the serum level of antioxidant enzymes. This change was in the direction of the normal value when compared with the group fed on soyabean only (Table 3).

**Table 2:** Chemical composition of extra virgin (EV) olive oil and genetically modified (GM) soyabean.

Component	Value
*EV Olive oil*	
Protein	0 mg
Fat	98.9 mg
Vitamin E	14 mg
*GM Soyabean*	
Moisture	88.1%
Ash	0.6%
Fiber	0.8%
Protein	1.5%

**Table 3:** Mean values of serum antioxidant enzymes in rats fed diets containing soyabean, olive oil or their combination (*n* = 10). Significant at *p* < 0.05 using ANOVA test a, b, c,… Significantly different between two comparison groups within the same litter and column using Duncan Multiple Range test at *p* < 0.05.

Group	Conc.	Lipid peroxidation mmol/L	Glutathione transferase mg/mL
Control -*ve* (negative)	-	4.379 ± 0.156 ^a, c^	14.7 ± 0.78 ^a, c^
GM soyabean	15%	5.04 ± 0.128 ^a^	12.846 ± 0.115 ^a^
EV olive oil	30%	4.398 ± 0.132 ^a, c^	14.604 ± 0.44 ^a, c^
GM soyabean + EV olive oil	15% + 30%	4.64 ± 0.35 ^a, c^	13.546 ± 0.115 ^a, c^

Diet effects on DNA: Maintaining rats on EV olive oil at concentration 30% for 65 days revealed a significant increase in the amount of DNA (ng/g of spleen) when compared with the control group. The amount of DNA (ng/g of spleen) significantly decreased when feeding rats GM soyabean (15%), while it increased significantly in rats fed EV olive oil (30%) and GM soyabean (15%) (Table 4).

**Table 4:** Effect of E.V.olive oil and G.M. soyabean on DNA (ng/g spleen). Significant at *p* < 0.05 using ANOVA test a, b, c,… Significantly different between two comparison groups within the same litter and column using Duncan Multiple Range test at *p* < 0.05.

Group	Conc./100g. ration	DNA
Control -*ve*	-	0.35 ± 0.04 ^a^
GM soyabean	15%	11.57 ± 0.58 ^d^
EV olive oil	30%	4.10 ± 0.22 ^b^
GM soyabean + EV olive oil	15% + 30%	5.74 ± 0.23 ^c^

Diet effects on cytogenicity: Feeding rats GM soyabean only caused a significant increase in micronucleated polychromatic erythrocytes (MPCEs) and the ratio of polychromatic to normochromatic erythrocytes (PCE/NCE). However, maintaining rats on ration mixed with EV olive oil and GM soyabean, instigated significant decrease of MPCEs and the ratio of PCE/NCE, though normochromatic erythrocytes (NCE) increased significantly when compared to the group fed soyabean only (Table 5).

**Table 5:** Effect of E.V. olive oil and G.M. soyabean on percentage of nucleated polychromatic erythrocytes (MPCE) and PCE/NCE ratio in rats (*n* = 10). Significant at *p* < 0.05 using ANOVA test a, b, c,… Significantly different between two comparison groups within the same litter and column using Duncan Multiple Range test at *p* < 0.05.

Group	Conc.	PCE	MPCE/1000	NCE	PCE/NCE
Control -*ve*	-	5000	5.60 ± 0.26 ^a^	2183 ± 31.89 ^g^	2.33 ± 0.4 ^a^
GM soyabean	15%	5000	4.45 ± 0.79 ^a^	1419 ± 36.05 ^d^	2.57 ± 0.22 ^a^
EV olive oil	30%	5000	9.11 ± 0.09 ^d^	829.5 ± 32.17 ^a^	6.18 ± 0.32 ^d^
GM soyabean + EV olive oil	15% + 30%	5000	8.00 ± 0.6 ^c^	1071 ± 19.76 ^c^	4.83 ± 0.3^c^

## 4. Discussion

EV olive oil is known to have a wide variety of benefits on many different body systems. This study was carried out to examine the effects of EV olive and G.M. soybean oil on DNA, as well as serum antioxidant enzymes.

In our study, rats were fed EV olive oil, GM soya bean, or a combination of the two, at concentrations (30%) or (15%). The study was conducted over 65 consecutive days.

We report a significant decrease in the level of lipid peroxidation and a significant increase in the level of glutathione dehydrogenase, when comparing the EV olive oil and GM soyabean combined group with GM soyabean fed animals only. These results may be attributed to the presence of antioxidants in EV olive oil, which has shown a number of positive effects on liver regeneration [21,22,23].

In addition, EV olive oil contains antioxidants and flavonoids that usually improve nutritional status and also has anti-inflammatory effects which may help in liver renewal [24,25,26].

Our data illustrates that the group of rats fed a mixed ration with EV olive oil for 65 successive days, showed a significant increase in the amount of DNA ng/g of spleen, when compared with the group maintained on soyabean alone.

On the other hand, tests on the rats fed GM soyabean alone revealed a significant decrease in the amount of DNA ng/g of spleen; while the group that was fed a mixed ration of EV olive oil and GM soyabean showed a significant increase in the amount of DNA ng/g of spleen; with these changes in parameters appearing to fall into the normal ranges.

The mechanism for the decrease in the amount of DNA was discussed by Zhou *et al.* [27] who reported on hepatocellular degeneration, necrosis, DNA damage and the lesions of the extracellular matrix induced by CCl4. Moreover, Morishita *et al.* [28], Amel *et al.* [23] and Vanitha *et al.* [9] showed that CCl4 could cause liver toxicity due to DNA strand breaks in hepatocytes.

Our results are in agreement with Salvini *et al.* [29], Jacomclli *et al.* [30] and Quiles *et al.* [31] who reported that a diet enriched with olive oil phenolic compound has a protective effect against DNA damage. This is in line with Fabiani *et al.* [32] who stated that olive phenolic compounds showed DNA oxidative preventive activity.

Fabiani *et al.* [32] suggested that EV olive oil has a protective activity against cancer cells. He described how cell proliferation can become arrested during the cell cycle phases with a role for apoptosis in tumor cells and hydroxytyrosol also being demonstrated. This may suggest that EV olive oil does have some anticancer properties. In this regard Salvini *et al.* [29] reported a reduction of DNA damage caused by consumption of an EV olive oil rich in phenols, particularly hydroxytyrosol. Interestingly, Machowetz *et al.* [33] and Fabiani *et al.* [32] described that high consumption of olive oil in the Mediterranean diet protected DNA against oxidative damage and reduced cancer incidence.

The mechanism for the increase in DNA was explained by Lopez *et al.* [34] who illustrated that minor compounds from EV olive oil (mainly phenolics) were antioxidants, and that these performed a radical scavenging role. These minimized the amount of reactive oxygen species generated by fatty acidperoxidation; hence the DNA damage can be reduced with a lower lipid peroxidation.

Jeffery [35], Jee-Youn *et al.* [36], Aiad [37], Khataibeh *et al.* [38] and Tudisco *et al.* [39] showed that a GM soya bean diet could lead to liver and/or DNA damage in rats. Our investigation revealed that feeding rats on ration mixed with EV olive oil showed significant increase in normochromatic erythrocytes (NCE). On the other hand, ratio of polychromatic to normochromatic erythrocytes (PCE/NCE) did not induce changes compared to the control group.

Sekene *et al.* [40] concluded that the protective effect of EV olive oil and sage against acrylamide mutagenicity may be due to the inclusion of antioxidant compounds and may be useful when added as a food additive when cooking at higher temperatures.

Rats fed a mixed ration with GM soybean showed a significant increase in MPCEs and in the ratio of PCE/NCE; while a significant decrease in NCE was observed. Feeding EV olive oil in combination with GM soyabean to rats, instigated a significant decrease in MPCEs and in the ratio of PCE/NCE and this helped to normalise their values. Suzuki *et al.* [41] described that a significant increase in PCE/NCE ratio and enhancement of mitotic activity of bone marrow cells could be considered as a sign of toxicity and/or damage of some organs of the body.

Hanan *et al.* [42] reported that the administration of soyabean extract resulted in a dose and time related increase in some blood biochemical parameters and in particular an increase in the percentage of MPECs and the ratio of PCE/NCE.

The mechanism of reducing MPCEs and the ratio of PCE/NCE were explained by Tang *et al.* [43] who concluded the oleanolic acid has protective effects on liver mitochondria and the mechanisms underlying its protection may be related to its inhibition to the Ca^2+^ induced mitochondrial swelling, mitochondrial membrane depolarization and intra-mitochondrial Ca^2+^ release on mitochondrial permeability transition (MPT). Moreover Sivikova *et al.* [44] described that an insufficient confirmation of the genitoxicity of CCl4 and the protective effect of the antioxidants, respectively were seen in cultures with metabolic activation.

## 5. Conclusions

We can conclude that adding EV olive oil to the diet of rats appears effective in inhibiting oxidative damage and may act as a protective agent against chronic diseases such as liver fibrosis, hyperlipidemia and diabetes. In addition, EV olive oil may also have a protective function against carcinogenic processes. Further clinical studies are therefore required to determine whether the observations observed in our study translate to human conditions and illnesses.
